# Family-Based Behavioural Intervention Program for Obese Children: An Observational Study of Child and Parent Lifestyle Interpretations

**DOI:** 10.1371/journal.pone.0071482

**Published:** 2013-08-07

**Authors:** Marie Teder, Evalotte Mörelius, Maria Nordwall, Per Bolme, Joakim Ekberg, Elisabeth Wilhelm, Toomas Timpka

**Affiliations:** 1 Division of Health, Activity and Care, Department of Social and Welfare Studies, Faculty of Health Sciences, Linköping University, Norrköping, Sweden; 2 Division of Paediatrics, Department of Clinical and Experimental Medicine, Faculty of Health Sciences, Linköping University, Linköping, Sweden; 3 Paediatric Clinic, Vrinnevi Hospital, Norrköping, Sweden; 4 School of Life Sciences, Skövde University, Skövde, Sweden; 5 Division of Social Medicine and Public Health, Department of Medical and Health Sciences, Faculty of Health Sciences, Linköping University, Linköping, Sweden; Scientific Directorate, Bambino Hospital, Italy

## Abstract

**Background:**

Family-based behavioural intervention programs (FBIPs) against childhood obesity have shown promising results, but the mediating mechanisms have not been identified. The aim of this study was to examine changes in obese childreńs lifestyle habits during a 2-year FBIP according to their own and parents’ reports, the concordance between these reports and the correlations to change in post-intervention z-BMI.

**Methods:**

An observational study of 26 children (8.3–12.0 years) and their parents participating in a 2-year FBIP was performed. Weight and height were measured from baseline to 12 months after the end of the program. Eating habits and physical- and sedentary activity were reported separately by children and parents. Data were analysed with regard to concordance between parents’ and children’s reports and association between the lifestyle reports and change in z-BMI at the study endpoint using descriptive statistics and parametric and non-parametric tests.

**Results:**

According to both children’s and parents’ reports, the level of physical activity among the children had increased after the intervention as well as the agreement between the informants’ reports. According to the children, eating habits had improved, while the parents’ reports showed an improvement only with regard to binge eating. The concordance between children and parents regarding eating habits was slight to fair also after the intervention. No statistically significant associations between changes in lifestyle reports and changes in z-BMI were observed.

**Conclusions:**

Child and parent reports of physical activity were found to converge and display an improvement in a 2-year FBIP, while the reports on eating habits showed a more refractory pattern. Changes in concordance and agreement between children and parents reports did not correlate with weight reduction. Further methods development and studies of the processes during family-based interventions against childhood obesity are warranted.

## Introduction

Obesity among children is a global health problem [1], [2]. To decrease the short- and long-term consequences of childhood obesity, present evidence emphasizes the importance of addressing behavioural factors such as healthy eating, physical activity, and a sedentary lifestyle [3], [4]. However, the design and implementation of behavioural change interventions for obese children have been shown to be challenging [5]–[7]. Parental involvement in the treatment of their children’s obesity is one of the relatively few design elements that has been associated with a satisfactory intervention outcome [8]–[12]. However, why and how a family-based childhood obesity intervention works has still not been settled. To answer these questions, it is necessary to identify mediators of the intervention effect [13]. While both children’s and parents’ assessments of the child’s lifestyle habits are used during family-based obesity interventions, there is limited research on how such assessments compare to each other and relate to treatment outcomes. A study comparing child and parental reports on eating patterns among 6–12 year-old children in a community setting in the U.S. found that these did not concur on the presence or type of eating episodes engaged in by the children. Although parents reported that their children engaged in binge eating more often than their children reported binge eating themselves, the children reported that they engaged in compensatory behaviors more frequently than their parents’ reports indicated. There was no association between the childreńs report and body composition, but children to parents who reported binge eating were more overweight [14]. Also, the Canadian CLASS study performed in a community setting [15] showed only a low to fair agreement between the parent’s and children’s reports of physical activity and parent’s reports explained better variation in weight status. Moreover, children who reported more physical activity and less sedentary activity compared with their parent’s reports were more likely to be overweight or obese than children whose reports were in agreement with their parents. To counterbalance such discordances, family-based treatment for eating disorders incorporates techniques aimed at facilitation of therapeutic alliance [16]. In family-based treatment, the illness is externalized from the child and family, viewed as a disease, thereby reducing guilt or blame and promoting cohesion within the family. The therapy focuses on solutions rather than putative etiologies, and also on improved agreement about the tasks at hand [17]. However, studies investigating the alliance between child and parent during family-based childhood obesity interventions as displayed by the agreement about eating habits, physical activity and sedentary activity are scarce. The aim of this study was therefore to examine the lifestyle habits of obese children during a 2-year Family-based Behavioural Intervention Program (FBIP) according to child and parental reports, the agreement between these reports, and the correlations to change in body mass index (standardized for age and sex) (z-BMI) from baseline to 12 months after the intervention program.

## Methods

**Figure 1:**
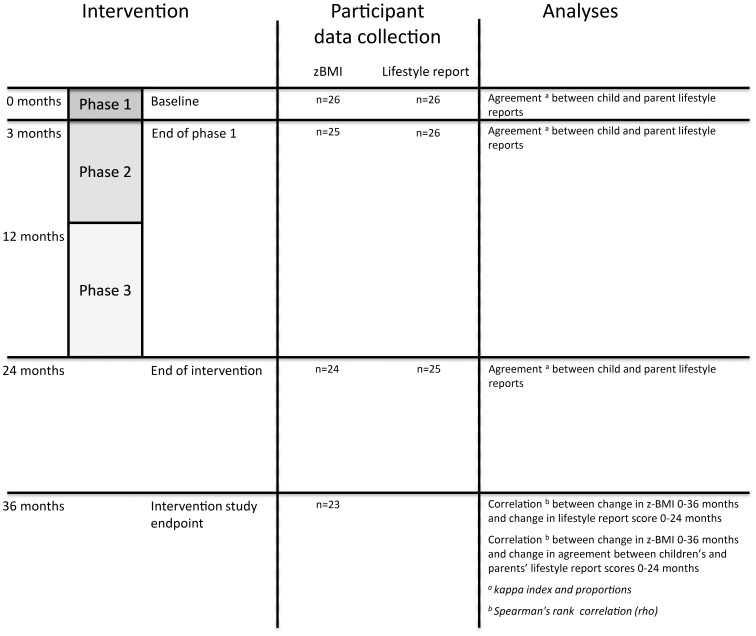
Overview of the study design.

An observational single-group design was used for a process evaluation study. The study was planned and reported according to the STROBE guidelines [18]. The primary end point measure was change in the child’s z-BMI 12 months after the intervention program compared with baseline z-BMI. Measures used for analyses of the intervention program were child and parental reports of the child’s lifestyle habits and agreement between the informants’ reports (Figure 1).

### Participants

Sixty-one children and their parents from two municipalities in southeast Sweden were assessed as obese by school nurses and referred to an FBIP in paediatric outpatient care. The inclusion criteria for the FBIP were age between 8– <12 years with obesity and absence of medical diseases [11]. Obesity was defined according to the International Obesity Taskforce (IOTF) criteria (above age- and sex-specific cut-offs corresponding to adult BMI, calculated as weight in kilograms divided by the square of height in meters, ≥30 kg/m^2^) [19]. Of 61 eligible families, 10 declined to participate and 51 were interviewed for suitability. The family’s motivation to change lifestyle habits and to participate in group sessions was discussed at the interview. The FBIP could only include around 24 children (3 groups of about 8 children each) and their parents. Families were excluded if the tutors evaluated them to not be sufficiently motivated or capable to complete the 2-year program. Twenty-six children, 14 boys and 12 girls, and their parents were finally included. After 3 months, one family withdrew from the group sessions [11].

### Intervention

The FBIP was based on a manual for a series of tutor-supervised group sessions for children with obesity and their parents [20]. Four tutors were paediatric registered nurses and two were dieticians. The tutors adhered strictly to the manual. Three groups of children and three corresponding groups of parents were assembled. The child and the parents from the same family were supported by the same two tutors. The programme started in 2004 and ended in 2006 and contained cognitive and behavioural components. During the first intensive phase, the groups met once a week for 3 months. Group sessions were held once a month throughout the second phase (months 4–12) and once every 3 months during the third phase (months 13–24). The second and third phases were maintenance phases. The sessions for the children lasted for 2 h, and included a light meal; the parent sessions lasted for 1.5 h. The sessions included discussions on how to support healthy eating habits, motivate the children to participate in regular physical activity and maintain these changes. The sessions also included discussions on methods to handle stress and dissatisfaction, solve problems, and find alternative ways to satisfaction. The parents were encouraged to help their children with two small realistic changes connected to nutrition and physical activity until the next session. The families were expected to record the child’s eating and physical activity in a diary [11].

### Data Collection

Eating habits, physical activity and sedentary activity were assessed by structured interviews adapted for children and for parents [21]. The interviews were performed at the same place as the group sessions and were conducted at baseline, 3 months and 24 months after the start of the intervention program; the child and his/her parents were interviewed separately. At baseline, all interviews were performed by the tutors and thereafter by independent researchers. The interview consisted of the following questions to assess eating habits: ‘Do you feel hungry most of the time?’ (yes/no), ‘Do you eat frequently between meals?’(yes/no), and ‘Do you sometimes eat a lot of food?’ (yes/no). The following questions were used to assess physical activity: ‘How many minutes/hours do you exercise per day (playing, cycling, walking up stairs, helping at home, etc.)?’ (<30 min/day, 30–60 min/day, 60–120 min/day, ≥2 h/day). The following questions were used to assess sedentary activity: ‘How many minutes/hours per day do you sit in front of the television, video, or computer?’ (<30 min/day, 30–60 min/day, 60–120 min/day, 2–3 h/day, ≥3 h/day). The interviewer categorized the respondent’s response into one alternative. To ensure validity, when the interviewer was hesitant about which response alternative to choose, the tentative choice was read back to the respondent to provide an opportunity to correct the category.

Weight and height were measured at baseline, and 3, 12, and 24 months after the start of the intervention, and 12 months after the end of the program (36 months) [11]. To compensate for age and sex variations, z-BMI was calculated using Swedish national reference values for children and a Box transformation formula [22].

### Statistical Analysis

For the descriptive computation of data from the child and parental reports, the reports on lifestyle habits were coded using binary variables (yes/no). The question ‘How many minutes/hours do you exercise per day?’ was dichotomized to ‘Physical activity ≥1 hour per day’ (yes/no). The question ‘How many minutes/hours per day do you sit in front of the television, video, or computer?’ was dichotomized to ‘Television, video, computer ≥1 hour per day’ (yes/no).

The Cochrane test was used to analyse if there were any changes between the children’s and the parent’s responses at baseline, and after 3 and 24 months; the McNemar test was used for post hoc analysis. The strength of agreement between the child and parental reports of the child’s habits about eating, physical activity and sedentary activity were analysed as concordance using Cohen’s kappa coefficient (κ). Cohen’s kappa represents the proportion of agreement corrected for chance; perfect agreement if κ = 1 [23], almost perfect if κ = 0.81–1.00, substantial agreement if κ = 0.61–0.80, moderate agreement if κ = 0.41–0.60, fair agreement if κ = 0.21–0.40, slight agreement if κ = 0.00–0.20, no better than chance agreement if κ = 0, and poor agreement if κ<0.00 [24]. These divisions are arbitrary but they do offer useful targets [23]; therefore proportions have been used as a complement. Positive agreement is the proportion of agreements among all positive reports and negative agreement is the proportion of agreements among all negative reports.

Spearman’s rank correlation analysis was used in the analytic computation of data on changes between the child and parental reports and changes in the primary outcome measure z-BMI at 36 months. For these computations, the original coding of the lifestyle variables was used (yes/no for eating habits, four levels for physical activity and five levels for sedentary activity). In addition, changes in agreement regarding these lifestyle variables during treatment were analysed to see if they were mediators of weight reduction at 36 months compared with baseline. The new change variables for lifestyle and agreement were recorded in three ascending levels of change: worse, unchanged and improved. A positive rho value indicates that a change in level for the worse gives more reduction in z-BMI and level improvement gives less reduction. Negative rho value indicate the opposite, i.e. a level change for the worse indicates less reduction in z-BMI and level improvement indicates more reduction.

SPSS Statistics Version 19 was used for the analysis. Tests were interpreted as statistically significant if *p*<0.05.

### Ethics Statement

The research protocol for the study was approved by the Regional Ethics Committee for research with human subjects at Faculty of Health Sciences, Linköping University, Sweden (dnr. 03–600) according to the World Medical Association Declaration of Helsinki 2002 [25]. Before the interviews, the parents were provided with written information about the purpose of the intervention and the interviews. They were asked to inform their child about the study and thereafter for written consent on their own and their child’s behalf if they decided to participate. The parents were also informed about that their own and their child’s participation was voluntary and the possibility of withdrawing at any time.

## Results

All children and parents participating in the intervention provided data at baseline and after 3 and 24 months except for the parents of one child who declined to participate after 24 months. Also, the family that withdrew after 3 months provided data after 24 months.

### Eating Habits

According to the children’s reports, all three eating habits had improved after 3 months of FBIP and the improvements were maintained after 24 months (Table 1). According to the parents’ accounts, improvement occurred and was maintained only regarding the children’s binge eating. Concordance between the child and parental reports on whether the children felt hungry and their binge eating habits had increased after the intervention program compared with baseline. The agreement measured as proportions showed a similar pattern.

**Table 1:** Child and parental assessments of the child’s eating habits and their agreements at baseline, after 3 months, and after 24 months of intervention program; concordance is displayed by Cohen’s kappa and agreement proportions as percentages.

	Child response								
	Baseline			3 months			24 months		
	Yes	No	Total	Yes	No	Total	Yes	No	Total
**Feeling hungry most of the time**									
Parental response									
Yes	5	4	9	0	2	2*	2	4	6
No	7	10	17	3	20	23	1	18	19
Total	12	14	26	3*	22	25	3*	22	25
Kappa		0.13			–0.11			0.34	
Agreement (%)		58			80			80	
Positive agreement (%)		48			0			44	
Negative agreement (%)		65			89			89	
**Frequent eating**									
Parental response									
Yes	14	2	16	5	1	6*	4	8	12
No	3	7	10	4	16	20	0	13	13
Total	17	9	26	9*	17	26	4*	21	25
Kappa		0.59			0.54			0.34	
Agreement (%)		81			81			68	
Positive agreement (%)		85			67			50	
Negative agreement (%)		74			86			79	
**Binge eating**									
Parental response									
Yes	8	7	15	0	4	4*	1	3	4*
No	5	6	11	2	20	22	2	19	21
Total	13	13	26	2*	24	26	3*	22	25
Kappa		0.08			–0.11			0.17	
Agreement (%)	54			77			80		
Positive agreement (%)	57			0			29		
Negative agreement (%)		50			87			88	

Statistically significant differences (*p*<0.05; McNemar Test) from baseline in respective group’s assessments are indicated (*).

### Physical Activity

The children’s reports indicated that the time they spent on general physical activity (e.g. playing, walking and cycling) had increased after both 3 and 24 months compared with baseline (Table 2). The parents’ accounts showed a similar pattern. After the end of the intervention program, agreement (concordance and proportions) between the children and the parents about the children’s level of physical activity had improved.

**Table 2:** Child and parental assessments of the child’s physical activity habits and their agreements at baseline, after 3 months, and after 24 months of intervention program; concordance is displayed by Cohen’s kappa and agreement proportions as percentages.

	Child response								
	Baseline			3 months			24 months		
	Yes	No	Total	Yes	No	Total	Yes	No	Total
**Physical activity ≥1 hour per day**									
Parental response									
Yes	5	8	13	22	2	24*	22	0	22*
No	6	7	13	1	0	1	1	2	3
Total	11	15	26	23*	2	25	23*	2	25
Kappa		–0.08			–0.06			0.78	
Agreement (%)		46			88			96	
Positive agreement (%)		42			94			98	
Negative agreement (%)		50			0			80	

Statistically significant differences (*p*<0.05; McNemar Test) from baseline in respective group’s assessments are indicated (*).

### Sedentary Activity

At baseline, most of the children and the parents reported that the children spent at least 1 hour per day on sedentary activity, such as watching television and playing video games (Table 3). According to both the children and their parents, there was no statistically significant change in the proportion of children with sedentary habits during or after the program. However, the agreement on sedentary activity (concordance and proportions) had improved during and after the intervention program.

**Table 3:** Child and parental assessments of the child’s sedentary activity habits and their agreements at baseline, after 3 months, and after 24 months of intervention program; concordance is displayed by Cohen’s kappa and agreement proportions as percentages.

	Child response								
	Baseline			3 months			24 months		
	Yes	No	Total	Yes	No	Total	Yes	No	Total
**Television, video, computer ≥1 hour per day**									
Parental response									
Yes	16	6	22	14	3	17	21	1	22
No	3	1	4	3	5	8	1	2	3
Total	19	7	26	17	8	25	22	3	25
Kappa		−0.02			0.45			0.62	
Agreement (%)		65			76			92	
Positive agreement (%)		78			71			95	
Negative agreement (%)		18			62			67	

Statistically significant differences (*p*<0.05; McNemar Test) from baseline in respective group’s assessments are indicated (*).

### Behavioural Change, Change in Child–parent Agreement and Change in z-BMI (0–36 Months)

Analysis of the association between changes in the reports of behaviour and change in z-BMI at 36 months showed no statistically significant correlations for children or parents (Table 4). No statistically significant correlations between change in agreement and change in z-BMI between baseline and at 36 months were observed (Table 5).

**Table 4:** Correlations between change in z-BMI from 0 to 36 months and change in children’s and parents’ reports of the child’s behaviour and habits from 0 to 24 months.

Reporting area	Correlation with z-BMI change from 0 to 36 months			
	Child report		Parent report	
	Rho (*p* value)	*n*	Rho (*p* value)	*n*
Feeling hungry most of the time	0.28 (0.20)	23	−0.08 (0.72)	23
Frequent eating	0.07 (0.76)	23	−0.33 (0.14)	22
Binge eating	0.14 (0.52)	23	0.11 (0.63)	22
Physical activity	0.29 (0.17)	23	−0.35 (0.11)	22
Sedentary activity	–0.06 (0.79)	23	0.01 (0.97)	22

**Table 5:** Correlations between change in z-BMI from 0 to 36 months and change in agreement of children’s and parents’ reports of the child’s behaviour and habits from 0 to 24 months.

	Correlation with z-BMI change from 0 to 36 months	
Reporting area	Rho (*p* value)	*n*
Feeling hungry most of the time	0.01 (0.98)	22
Frequent eating	–0.15 (0.51)	22
Binge eating	–0.28 (0.21)	22
Physical activity	–0.34 (0.12)	22
Sedentary activity	–0.30 (0.17)	22

## Discussion

We set out to examine the lifestyle habits of obese children during a 2-year FBIP according to their own and parents’ reports, the agreement between these reports, and the correlations to change in z-BMI from baseline to 12 months after the intervention program. The level of physical activity among the children had increased according to the children and the parents, but no decrease in sedentary activity had been accomplished according to both groups of informants. Post-program concordance regarding physical and sedentary activities had improved to perfect. According to the children, their eating habits had improved after the intervention program; the parents’ reports only indicated an improvement with regard to binge eating. Concordance between children and parents regarding eating habits remained between slight to fair after the intervention. We found no statistically significant associations between changes in the lifestyle variables or concordance and changes in z-BMI.

After the intervention, 92% of the children and 88% of the parents reported that the children participated in physical activities ≥1 hour per day. This activity level is in accordance with both the World Health Organization [26] and Swedish recommendations of at least 60 minutes of physical activities every day [27]. The agreement between child and parental reports had also improved substantially for both physical activity and sedentary activity. These high concordances can be interpreted as a positive output of the FBIP in light of the CLASS study [15], where concordance between children’s and parents’ reports about physical activity was found to be associated with normal weight. In contrast, we found that eating habits had according to child self-reports improved after the intervention, while the parental reports only showed a decrease in binge eating. These discrepancies between children’s and parents’ reports on eating behaviours are in line with previous studies [28]. One important source of bias could be that the children were affected by shame and responded according to what they thought that they were expected to say [29]. For the parents, a major difficulty in assessing their child’s eating habits could be the lack of constant presence in their child’s life, especially when the child gets older and more independent [30], [31]. In addition, the disagreement between child and parental behavioural reports could depend on the children’s ability to understand some concepts [15]. In the present study, the young age of the children at baseline could have made it more difficult for them to assess and respond to the questions.

These observations lead to the more general issue of assessing children’s lifestyle behaviour during FBIPs. In this study, the data on lifestyle behaviors were primarily collected from children and parents for use in the intervention process. The only consistently observed positive change of behaviour associated to the program was regarding physical activity, both according to the informants’ reports and their concordance. One explanation of these findings is the relative overtness of physical activity assessments, where also pedometers and other technical devices rather straight-forwardly can be used for passive surveillance [32]. However, corresponding possibilities for corroborated monitoring of eating behaviours are not available. It is also generally hard to assess subjective sensations, e.g. if a child feels hungry. Although a previous study reported that diaries can help improve eating habits in families with obese children [33], the diary used in the current study was not sufficient to reach concordance regarding reports of the child’s eating habits. One possibility to improve the validity of eating behaviour reports among children is to concurrently collect, combine, and analyse reported and monitored data on energy intake and energy expenditure using amalgamations of electronic surveillance methods [34].

Previous studies have indicated that cohesion and agreement within families during family therapy is beneficial with regard to achieving treatment goals [35], [36]. In this study, we did not observe any statistically significant association between concordance between children’s and parents’ reports and reduction of z-BMI. There are several possible explanations for this finding. The FBIP was organized in separate groups for children and parents, which may have led to that other supportive alliances than that between child and parent were more endorsed, e.g. between therapist and child or between the children. Moreover, it was for organizational reasons only possible to include about 25 families in the 2-year FBIP. Therefore, we did not perform any power calculation for the size of the study population or as a basis for statistical testing. An evident risk for type 2 errors must thus be taken into account when interpreting the results from analyses of association between concordance and treatment outcome.

This non-randomized study has strengths, but also several evident limitations. The main strengths are the long-term follow-up period and the low non-participation rate. The foremost limitation is the single-group design, having as consequence that it is not possible to distinguish between FBIP effects and normal cognitive and behavioural maturation and development in childhood. The items used for life-style assessments during the treatment process were derived from the manual for tutor-supervised group sessions used for the FBIP [20], and the data were used in the intervention process. The mentioned weaknesses of within-intervention assessments of lifestyle behaviours among children are thereby pertinent also when interpreting the study results. Although being clinically evaluated for face validity, the construct validity of the items was not formally analyzed. Also, the data were re-categorized into a dichotomized format in order to simplify the analysis model, which reduced the variability and may have influenced the validity of the results. We used both concordance and proportions to describe agreement between children and their parents. Attaining a perfect kappa score requires balanced marginals, meaning that the proportions in the table have a presumed equal distribution. If this is not the case, Cicchetti and Feinstein [37] recommend calculating positive and negative agreements, which are analogous to sensitivity and specificity. In this study, the results showed high agreement and low kappa scores, indicating that the tables contained unbalanced margins [38]. Therefore, positive and negative agreements were calculated.

We conclude that the association between child and parental reports of lifestyle habits and factual behaviour and weight outcomes during FBIPs against childhood obesity is complex. Although child and parental reports of physical activity during the intervention were found to converge and display an improvement, the reports on eating habits showed a more divergent pattern. No association between any of these patterns and weight reduction was observed. These findings are also concordant with that conducting conjoint family therapy is not simply a matter of treating young people in the presence of their parents, but that a systemic perspective is essential for understanding how family members are reacting to what goes on [39]. Method development and further study of the processes mediating the effect during FBIP programmes against childhood obesity are warranted.
